# Transmission risk of COVID-19 in high school and college water polo

**DOI:** 10.1186/s12879-022-07448-6

**Published:** 2022-05-11

**Authors:** Raymond J. Kreienkamp, Christopher J. Kreienkamp, Cindy Terrill, Mark E. Halstead, Jason G. Newland

**Affiliations:** 1 Department of Pediatrics, St. Louis Children’s Hospital, Washington University School of Medicine, 620 S. Taylor Ave, Northwest Tower 10113, St. Louis, MO 63110 USA; 2 Department of Electrical Engineering, University of Notre Dame, Notre Dame, IN USA; 3 Division of Infectious Diseases, St. Louis Children’s Hospital, Washington University School of Medicine, St. Louis, MO USA; 4 Department of Orthopedic Surgery, St. Louis Children’s Hospital, Washington University School of Medicine, St. Louis, MO USA

**Keywords:** Water polo, COVID-19, Transmission, NFHS, NCAA

## Abstract

**Background:**

Concerns that athletes may be at a higher risk for severe acute respiratory syndrome coronavirus 2 (SARS-CoV-2) transmission has led to reduced participation in sports during the COVID-19 pandemic. We aimed to assess COVID-19 incidence and transmission during the spring 2021 high school and college water polo seasons across the United States.

**Methods:**

This prospective observational study enrolled 1825 water polo athletes from 54 high schools and 36 colleges. Surveys were sent to coaches throughout the season, and survey data were collected and analyzed.

**Results:**

We identified 17 COVID-19 cases among 1223 high school water polo athletes (1.4%) and 66 cases among 602 college athletes (11.0%). Of these cases, contact tracing suggested that three were water polo–associated in high school, and none were water polo–associated in college. Quarantine data suggest low transmission during water polo play as only three out of 232 (1.3%) high school athletes quarantined for a water polo–related exposure developed COVID-19. In college, none of the 54 athletes quarantined for exposure with an infected opponent contracted COVID-19. However, in both high school and college, despite the physical condition of water polo athletes, both high school (47%) and college athletes (21%) had prolonged return to play after contracting COVID-19, indicating the danger of COVID-19, even to athletes.

**Conclusions:**

While COVID-19 spread can occur during water polo play, few instances of spread occurred during the spring 2021 season, and transmission rates appear similar to those in other settings, such as school environments.

**Supplementary Information:**

The online version contains supplementary material available at 10.1186/s12879-022-07448-6.

## Introduction

Concerns of sports-associated severe acute respiratory syndrome coronavirus 2 (SARS-CoV-2) transmission has led to a reduction in sports participation during the COVID-19 pandemic. These limitations forced cancellation of high school and college sport seasons across the United States and limited sports participation around the world. For youth, these sports restrictions led to significant effects on mental and physical health [1, 2]. As a result, many advocated to restart sports in a manner that still minimized the risk of contracting COVID-19.

To facilitate restarting sports, governmental organizations risk stratified sports from those thought least and most likely to facilitate COVID-19 spread. Sports deemed less likely to facilitate transmission were allowed to occur with few restrictions, whereas those deemed riskier faced more severe restrictions. The Centers for Disease Control and Prevention (CDC) highlighted numerous factors contributing to sport risk, including physical distancing, mask usage, hand hygiene, and respiratory etiquette [3]. However, these guidelines made it difficult to risk stratify some sports, such as water polo, where masks cannot be used, but play in an aquatic and, oftentimes, chlorinated environment. As a result, recommendations for water polo varied widely. The Italian National Olympic Committee suggested water polo was among the safer sports, ranking its risk similar to beach volleyball, whereas the state of California classified it as high risk and imposed severe restrictions [4, 5]. As a result, due to lack of data, governments were forced to make recommendations that potentially overestimated or underestimated risk.

In spring 2021, both high school and college water polo resumed to levels beyond what was previously permitted in the COVID-19 pandemic. Before the pandemic, greater than 40,000 youth participated in high school state-sanctioned water polo, with most in California [6]. Seasons vary by geographic area across the academic year. In spring 2021, high school state-sanctioned water polo began in nine states, all of which are represented in this survey. Total athlete number was significantly reduced given local regulations in many areas. Normally, there are over 100 combined men’s and women’s NCAA water polo teams, with men participating in fall and women in spring. None had been allowed to compete in fall 2020. However, in spring 2021, 24 men’s teams participated in an abridged season, and a select number of women’s teams were allowed to participate. While overall athlete numbers were reduced from typical seasons, this served as a perfect environment to monitor COVID-19 transmission and risk with water polo with still substantial participation, from across the country, at multiple age levels. By polling coaches at both the high school and college levels, we were able to gather the first quantifiable data of water polo risk with COVID-19. The aim of this study was therefore to determine COVID-19 transmission risk associated with water polo.

## Methods

**Fig. 1:**
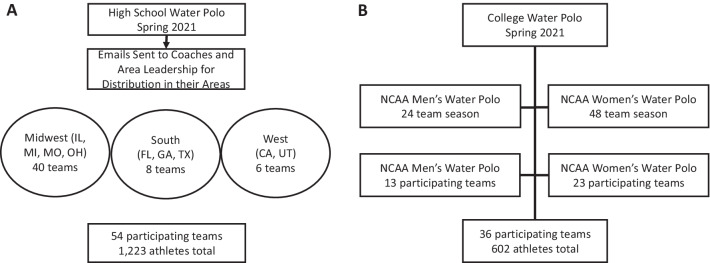
Overview of teams participating in the study at both the (**A**) high school and (**B**) college level

Study data (Fig. 1) was collected using two sets of surveys (Additional file 1: Appendix S1), one for high school and one for college, using REDCap electronic data capture tools hosted at Washington University School of Medicine [7, 8]. REDCap (Research Electronic Data Capture) is a secure, web-based software platform designed to support data capture for research studies, providing (1) an intuitive interface for validated data capture; (2) audit trails for tracking data manipulation and export procedures; (3) automated export procedures for seamless data downloads to common statistical packages; and (4) procedures for data integration and interoperability with external sources. Institutional review board (IRB) approval for use of surveys was granted (IRB #202102119).

High school coaches were informed of the study by water polo leadership in their area. Coaches interested in enrolling filled out the “General Information” survey. Every 2 weeks throughout the season, coaches were emailed the “14 Day Survey” to the email specified to collect data on team activities over the previous 2 weeks. At the end of the season, a “Final Survey” was sent to coaches. High school coaches were responsible for filling out survey, and they would receive a repeat email in 48 h had data not been submitted.

College coaches were informed of the study by email after the conclusion of their seasons. All coaches interested in participating were able to complete the “College Water Polo” survey. The college coaches submitted the data themselves. Data from these surveys were collected in REDCap and then analyzed.

Surveys were developed with the input of the co-authors to gather information helpful for understanding COVID-19 transmission risk with water polo. All surveys were sent for review to select coaches to ensure they were interpreted as intended. The high school survey was assessed longitudinally across the season so that real time data could be gathered.

Athlete exposures refers to one game or practice for one athlete. This was calculated using number of athletes, number of games, and number of practices. At the high school level, these data were collected on a bi-weekly basis, and the sum of athlete exposures was calculated for the entire season.

Contact tracing was primarily performed by dedicated personnel at each school. In high school, school districts were governed by protocols for contact tracing based on the rules of their local health departments. Local health departments, at times, also assisted with contact tracing. The NCAA required a school-designated representative to perform contact tracing. For some NCAA schools, contact tracing did not clearly determine if COVID-19 spread inside or outside of water polo play. In those cases not discriminated by school, coaches were asked to decide, if able, on if transmission occurred as a result of water polo activities.

## Results

### High school water polo

Fifty-four high school teams, with a total of 1223 high school athletes, from 9 different states (Florida, Georgia, California, Illinois, Michigan, Missouri, Ohio, Texas, Utah), enrolled in the study (Table 1). The spring high school season began as early as 1/4/21, and ended as late as 6/10/21. Before the season started, 33 of 1223 athletes (2.7%) had contracted COVID-19. During the high school season, 17 total COVID-19 cases were identified, indicating an incidence of 1.4%. Most of these cases were unrelated to in-pool activity. A total of 232 athletes were quarantined for close COVID-19 contacts during water polo practices or games. Quarantine criteria varied by health department and school district, though were reflective of overall CDC guidelines at the time [9]. Local health departments, as well as school officials, were engaged in close contact tracing of positive contacts. Of those athletes quarantined, only 3 (1.3%) tested positive for COVID-19, indicating very low transmission during water polo activities.

**Table 1:** COVID-19 in high school water polo

	Boys *n* (%)	Girls *n* (%)	Co-Ed *n* (%)	Overall *n* (%)
Total athletes	421	500	302	1223
Teams	17	23	14	54
Athlete exposures^a^	15,049	21,167	14,091	50,307
Total COVID-19 cases (percentage of total athletes who contracted COVID-19)	6 (1.4%)	9 (1.8%)	2 (0.6%)	17 (1.4%)
Cases per 10,000 athlete exposures^a^	4.0	4.2	1.4	3.4
Number of athletes requiring > 7 days after quarantine end to return to full activity	4 (66%)	3 (33%)	1 (50%)	8 (47%)
Total quarantines for water polo contacts	70	136	26	232
Total positives from water polo quarantines	2 (2.9%)	1 (0.7%)	0 (0%)	3 (1.3%)

^a^Athlete exposures refers to one game or practice for one athlete

Throughout the season, teams performed mitigation strategies to decrease COVID-19 spread (Table 2). No statistical differences were found in COVID-19 rates with these strategies given the overall low incidence. While widespread routine COVID-19 testing is certainly helpful, only one COVID-19 case was identified through this testing.

**Table 2:** High school water polo COVID-19 mitigation strategies

	Boys *n* (%)	Girls *n* (%)	Co-Ed *n* (%)	Total *n* (%)
Mask usage while on deck	15 (88%)	21 (91%)	14 (100%)	50 (93%)
Symptom screening before activity	15 (88%)	18 (78%)	8 (57%)	41 (76%)
Locker room usage	8 (47%)	7 (30%)	8 (57%)	23 (43%)
Outdoor pool location	5 (29%)	4 (17%)	1 (7%)	10 (19%)
Routine COVID-19 testing	4 (17%)	1 (2%)	1 (2%)	6 (11%)

### College water polo

After postponing the regularly scheduled men’s fall 2020 water polo season, the NCAA conducted a shortened men’s season in spring 2021, running from January until March 21, 2021. A normal women’s season was held, running from January 2021 until May 16, 2021.

Thirty-six teams enrolled in our study at the conclusion of their season (Table 3). This included 602 athletes, with 66 COVID-19 cases identified over the course of the season. As part of the NCAA protocols [10], all schools completed some form of routine COVID-19 testing, with most teams testing three times per week (Table 4). At all institutions, contact tracing was performed. Interestingly, none of the 66 cases were traced to in-pool contact, with the largest percentage traced to social gatherings (Table 5). In fact, numerous instances occurred where players were in close contact in the pool within 48 h of an individual developing COVID-19, but no cases were reported. In the women’s season, 54 athletes were identified who required quarantine after an opponent developed COVID-19 within 48 h of playing a game. In each of these cases, none of the 54 at-risk athletes developed COVID-19.

**Table 3:** COVID-19 in college water polo

	Men *n* (%)	Women *n* (%)	Overall *n* (%)
Total athletes	233	369	602
Teams	13	23	36
Total games	77	245	322
Athlete exposures^a^	11,214	24,748	35,962
Total COVID-19 cases	17 (7.3%)	49 (13.3%)	66 (11.0%)
Cases per 10,000 athlete exposures^a^	15.1	19.8	18.3
Number of symptomatic cases	8 (47%)	30 (61%)	38 (58%)
Number of athletes requiring > 7 days after quarantine end to return to baseline activity level	3 (18%)	11 (22%)	14 (21%)
Teams with cases	7 (54%)	9 (39%)	16 (44%)

^a^Athlete exposures refers to one game or practice for one athlete

**Table 4:** Frequency of mandatory team-wide COVID-19 surveillance testing in college water polo

Testing frequency	Men *n* (%)	Women *n* (%)	Total *n* (%)
Less than once per week	0 (0%)	1 (4%)	1 (3%)
Weekly	0 (0%)	1 (4%)	1 (3%)
Two times per week	2 (15%)	1 (4%)	3 (8%)
Three times per week	11 (85%)	15 (65%)	26 (72%)
Four times per week	0 (0%)	3 (13%)	3 (8%)
Daily	0 (0%)	2 (9%)	2 (6%)

**Table 5:** Source of college water polo COVID-19 infections as identified by contact tracing

	Men *n* (%)	Women *n* (%)	Total *n* (%)
In-water transmission	0 (0%)	0 (0%)	0 (0%)
Social gatherings	6 (35%)	30 (61%)	36 (54%)
Roommate	2 (12%)	11 (22%)	13 (20%)
Family	1 (6%)	3 (6%)	4 (6%)
Other	8 (47%)	5 (10%)	13 (20%)

Of the 66 cases of COVID-19 identified, 58% of athletes were symptomatic. In 21% of cases, athletes required more than 7 days after quarantine to return to their previous level of activity. Again, this highlights the dangerous nature of the virus, even in young athletes. While vaccination efforts are primarily intended to prevent severe disease, it is possible that they may have helped mitigate spread of COVID-19. Yet, more than 50% of teams had less than a 50% vaccination rate by the end of the season, which is below the threshold needed for herd immunity (Table 6). This vaccine rate is not surprising, since most did not have access to vaccines until late in their respective seasons. One case of COVID-19 occurred in an individual that had previously been vaccinated. As a result, our data suggest that COVID-19 transmission during water polo play is unlikely in most circumstances.

**Table 6:** College water polo vaccination rates at end of spring season

Percentage vaccinated	Men *n* (%)	Women *n* (%)	Total *n* (%)
< 25%	5 (38%)	7 (30%)	12 (33%)
26–50%	2 (15%)	7 (30%)	9 (25%)
51–75%	0 (0%)	4 (17%)	4 (11%)
76–99%	4 (31%)	5 (22%)	9 (25%)
100%	2 (15%)	0 (0%)	2 (6%)

## Discussion

Since the start of the COVID-19 pandemic, more is now known about the spread of COVID-19 and those environments conducive to spread. This is also true in athletics, where more data have emerged about COVID-19 in sport. Recently, multiple studies have demonstrated minimal disease transmission in soccer [11, 12]. Understanding more about disease spread in athletics is important to allow government organizations to advise how to proceed with sports in a safe manner.

This study is the first to monitor disease and disease transmission in water polo. Because of lack of studies to this point, recommendations have been disparate across the United States and around the world. While anecdotal reports suggested little spread and risk with water polo, this study quantifies this risk. Strikingly, our data suggests that COVID-19 risk through in-pool water polo activity, at both the high school and college level, is minimal. In fact, our data here notes that only 1.3% of high school athletes playing water polo turned positive after an in-pool exposure and no college athletes turned positive after such an exposure. Interestingly, two boys’ cases occurred after athletes were forced to leave an outdoor pool due to inclement weather. As athletes huddled together under a shelter, they did not wear masks, but the coaches did. Two players within 48 h of this event contracted COVID-19. As a result, it is uncertain whether these cases were due to in-pool contact or were related to lack of masks while huddled under the pavilion.

Remarkably, the 1.4% incidence overall during the high school water polo season is even less than the 2% positivity rate reported in schools after masked exposure [13]. Additionally, this rate is lower than the percentage of athletes who contracted COVID-19 before the season. While the incidence of COVID-19 was greater before the season, this might also suggest that water polo had some protective effect against COVID-19 given that coaches and athletes may have adhered more closely to protocols in order to ensure they remained eligible to compete in activities. Given these findings, protocols limiting stricter return to sports should be re-evaluated, particularly with the mental and physical health benefits they provide for athletes.

Why water polo, without masks and periods of close contact, is an inhospitable environment for COVID-19 transmission remains to be investigated. Some have suggested that the chlorinated environment, higher environmental temperature and humidity, and air circulation, whether indoors or outdoors, decrease the risk of COVID-19 spread [14]. A recent study demonstrated the rapid inactivation of SARS-CoV-2 in properly chlorinated pools [15]. However, our data suggest that lack of mask usage should not result in governmental organizations automatically assigning water polo the highest risk.

While risk mitigation techniques did not have a quantifiable effect in decreasing COVID-19 transmission, multiple coaches specifically mentioned that use of these techniques decreased spread. Given that 93% of teams in the high school cohort wore masks on deck, this may have prevented greater incidence of COVID-19 among team members.

Vaccination continues to be important in limiting the spread of COVID-19. As vaccinations become more widely available, those who have access should continue to vaccinate to further decrease risk. However, vaccines will not entirely prevent COVID-19, as evident in the one collegiate water polo athlete in this study who contracted COVID-19 even after vaccination. Nevertheless, given the significant symptoms among those who contracted COVID-19, including those who took longer than 1 week to return to play, vaccination should be an important effort for those interested in protecting athletes.

## Limitations

The weaknesses of this study include incomplete participation of athletes and teams competing during the spring 2021 season. While greater than 60% of high school teams in Missouri were included in this study and greater than 40% of college teams, including greater than 50% of men’s teams, many teams were unable to participate, either due to administrative prohibition or lack of knowledge of the study. However, we did survey over 1800 total athletes from a diverse geographic area. Given this diverse cohort, it should be representative of a broader population. More studies will be needed to continue to track COVID-19 in the water polo population.

In addition, all surveys were completed by coaches. This assumes complete knowledge of athlete health and contacts by coaches involved, which sometimes was performed by other staff at the school. Additionally, this data relies on external contact tracers to determine the source of infection. Our study also relied on schools to determine who was a high-risk contact needing quarantine. This introduced heterogeneity into the study that would have been prevented if one contact tracer with one set of standards would have performed contact tracing for all subjects. Numerous coaches remarked on the inconsistency in establishing quarantine criteria among neighboring schools and school districts. In some instances, schools may have been more conservative given the importance of slowing COVID-19 spread, thus overestimating the number of athletes who needed to quarantine and were a high-risk contact.

There is also risk of response bias with this survey. Coaches may have been interested in reporting numbers that showed little transmission amongst their program or supported the safety of water polo. Alternatively, coaches with significant spread may have failed to respond. The survey was anonymous to decrease this risk. Further, at outset of this survey, there was a concerted effort to identify any water polo transmission to inform the community of what might be done to minimize spread. However, over the course of the study, no significant water polo transmission was reported, also making us more confident that our results are reflective of the more general population.

It is also possible that some athletes may not have reported symptoms of COVID-19 in order to avoid quarantine and continue athletic participation. This may have prevented identification of some COVID-19 cases at the high school level, but not the college level, where routine testing was mandatory.

## Conclusion

Our cohort is the first to demonstrate the relative safety of water polo during the COVID-19 pandemic. The findings suggest that water polo may be played safely with proper mitigation techniques outside of the pool. Furthermore, governmental organizations should evaluate water polo risk classification with the data we have here—namely, that we see little transmission of COVID-19 with water polo, and risk is likely minimal with proper mitigation strategies. As more competition is allowed, more exposures will occur, providing a greater understanding of relationship between COVID-19 and water polo.

## Supplementary Information

**Additional file 1: Appendix S1.** Survey of water polo coaches—general information.

## Data Availability

The datasets generated during and analyzed during the current study are not publicly available given that public release of information was not authorized by participants nor approved by Institutional Review Board. Requests for further information should be directed to R. Kreienkamp.

## Abbreviations

CDC: Centers for Disease Control and Prevention
COVID-19: Coronavirus disease 2019
NCAA: National Collegiate Athletic Association
IRB: Institutional review board
SARS-CoV-2: Severe acute respiratory syndrome coronavirus 2
