# Supplementary material for: Transmission risk of COVID-19 in high school and college water polo
Source: BMC Infect Dis. 2022 May 11;22:450. doi: 10.1186/s12879-022-07448-6 (PMC9092321; doi:10.1186/s12879-022-07448-6)
Supplement: Supplementary file 1 — Additional file 1: Appendix S1. Survey of water polo coaches—general information. [file 12879_2022_7448_MOESM1_ESM.pdf]

Survey of Water Polo Coaches - General Information

Thank you for your willingness to participate in this study. By quantifying the effect of COVID-19 on high school water polo across the country, we will better understand risks, mitigation strategies, and other variables that influence the ability to play water polo safely.

Email ________________________________

High School ________________________________

City ________________________________

State	◯ AK<br>◯ AL<br>◯ AR<br>◯ AZ<br>◯ CA<br>◯ CO<br>◯ CT<br>◯ DE<br>◯ FL<br>◯ GA<br>◯ HI<br>◯ IA<br>◯ ID<br>◯ IL<br>◯ IN<br>◯ KS<br>◯ KY<br>◯ LA<br>◯ MA<br>◯ MD<br>◯ ME<br>◯ MI<br>◯ MN<br>◯ MO<br>◯ MS<br>◯ MT<br>◯ NC<br>◯ ND<br>◯ NE<br>◯ NH<br>◯ NJ<br>◯ NM<br>◯ NV<br>◯ NY<br>◯ OH<br>◯ OK<br>◯ OR<br>◯ PA<br>◯ RI<br>◯ SC<br>◯ SD<br>◯ TN<br>◯ TX<br>◯ UT<br>◯ VA<br>◯ VT<br>◯ WA<br>◯ WI<br>◯ WV<br>◯ WY
Team Participants	◯ Boys<br>◯ Girls<br>◯ Co-Ed
Pool Location	◯ Indoor<br>◯ Outdoor<br>◯ Both Indoor and Outdoor
Date of First Practice	______________________

Date of State Championship	__________________________________
Number of Athletes on JV Team	__________________________________
Number of Athletes on Varsity Team	__________________________________
Does your school perform routine frequent testing for COVID-19?	◯ Yes<br>◯ No
What type of tests does your school perform?	◯ Antigen<br>◯ PCR<br>◯ Antibody<br>◯ Not sure
How often does your school test?	◯ >1 per week<br>◯ Weekly<br>◯ 2-3 times per month<br>◯ Monthly
How many athletes on JV team have tested positive for COVID-19 in the last three months?	__________________________________<br>(Please leave blank if uncertain)
How many athletes on varsity team have tested positive with COVID-19 in the last three months?	__________________________________<br>(Please leave blank if uncertain)
Does your team mask while on deck and not in the pool?	◯ Yes<br>◯ No
Are you doing health screening, including taking temperatures, before each event (practice/games)?	◯ Yes<br>◯ No
Are you using locker rooms (to change before/after games or practices)?	◯ Yes<br>◯ No

Survey of Water Polo Coaches - 14 Day Survey

Thank you for your willingness to participate in this study. By quantifying the effect of COVID-19 on high school water polo across the country, we will better understand risks, mitigation strategies, and other variables that influence the ability to play water polo safely.

Thank you for your continued support of our study.

Team Participants

◯ Boys
◯ Girls
◯ Co-Ed

Number of Athletes on JV Water Polo Team

____

Number of Days with JV Practices And/Or Games In Last Two Weeks

____
(E.g. If you had three game days and four practice days, please say 7. If 10 practices, please say 10. If three games but no practices, please say 3. If you had two games on one day, please only count 1 for that day.)

Number of JV athletes quarantined in last two weeks due to contacts in water polo

____

Can you explain reason for quarantine?

____

Number of JV athletes in quarantine who tested positive for COVID-19

____
(Count the number of athletes who, after being placed in quarantine due to contact tracing associated with water polo, developed COVID-19. This includes those athletes who had been identified based on contact in water polo. This does not include individuals who were in quarantine based on contacts outside of water polo and then tested positive.)

Number of Athletes on Varsity Team

____

Number of Days with Varsity Practices And/Or Games in Last Two Weeks

____
(E.g. If you had three game days and four practice days, please say 7. If 10 practices, please say 10. If three games but no practices, please say 3. If you had two games on one day, please only count 1 for that day.)

Number of Varsity athletes quarantined in last two weeks due to contacts in water polo

____

Can you explain reason for quarantine?

____

Number of Varsity athletes in quarantine who tested positive for COVID-19

____
(Count the number of athletes who, after being placed in quarantine due to contact tracing associated with water polo, developed COVID-19. This includes those athletes who had been identified based on contact in water polo. This does not include individuals who were in quarantine based on contacts outside of water polo and then tested positive.)

Does your school require routine COVID-19 testing?

○ Yes
○ No

Number of JV COVID-19 tests performed since last survey

____

Number of positive JV COVID-19 tests since last survey

____
(New cases of COVID-19, not reported in previous weeks)

Number of Varsity COVID-19 tests performed since last survey

____

Number of positive Varsity COVID-19 tests since last survey

____
(New cases of COVID-19, not reported in previous weeks)

Survey of Water Polo Coaches - Final Survey

Thank you for your willingness to participate in this study. By quantifying the effect of COVID-19 on high school water polo across the country, we will better understand risks, mitigation strategies, and other variables that influence the ability to play water polo safely.

Thank you for your participation in our study this season.

Team Participants

- ◯ Boys
- ◯ Girls
- ◯ Co-Ed

How many JV games were played?

____

How many JV games were cancelled due to COVID-19?

____

How many days was the JV team completely shut down due to COVID-19 quarantines/infections?

____

(Please count each day that the entire JV team did not practice/play due to COVID-19 quarantines/exposures.)

Number of JV athletes at end of season

____

How many JV athletes overall contracted COVID-19?

____

(Please include total number of positive COVID-19 cases, including those cases not thought to be associated with water polo.)

How many of these cases were thought to be associated with water polo?

____

How do you believe transmission occurred during water polo?

____

(e.g. opponent had COVID-19; teammate had COVID-19; etc. If opponent had COVID-19, please specify if player who developed COVID-19 was guarding that individual? And, if known, at center? If teammate had COVID-19, if you had any information on if certain drills/practice activities were occurring when presumed exposure took place, that would be tremendously helpful.)

How many JV athletes, after contracting COVID-19, required greater than seven days after quarantine ended to return to full activity?

____

What percentage of your JV team was fully vaccinated by end of the season? (Fully-vaccinated means two weeks have elapsed since receiving the second dose of either the Pfizer or Moderna vaccine, or two weeks after receiving the Johnson & Johnson vaccine)	◯ Unsure<br>◯ 0%<br>◯ 1-25%<br>◯ 26-50%<br>◯ 51-75%<br>◯ 76-100%
How many Varsity games were played?	__________________________
How many Varsity games were cancelled due to COVID-19?	__________________________
How many days was the Varsity team completely shut down due to COVID-19 quarantines/infections?	__________________________<br>(Please count each day that the entire Varsity team did not practice/play due to COVID-19 quarantines/exposures.)
Number of Varsity athletes at end of season	__________________________
How many Varsity athletes overall contracted COVID-19?	__________________________<br>(Please include total number of positive COVID-19 cases, including those cases not thought to be associated with water polo.)
How many of these cases were thought to be associated with water polo?	__________________________
How do you believe transmission occurred during water polo?	__________________________<br>(e.g. opponent had COVID-19; teammate had COVID-19; etc. If opponent had COVID-19, please specify if player who developed COVID-19 was guarding that individual? And, if known, at center? If teammate had COVID-19, if you had any information on if certain drills/practice activities were occurring when presumed exposure took place, that would be tremendously helpful.)
How many Varsity athletes, after contracting COVID-19, required greater than seven days after quarantine ended to return to full activity?	__________________________
What percentage of your Varsity team was fully vaccinated by end of the season? (Fully-vaccinated means two weeks have elapsed since receiving the second dose of either the Pfizer or Moderna vaccine, or two weeks after receiving the Johnson & Johnson vaccine)	◯ Unsure<br>◯ 0%<br>◯ 1-25%<br>◯ 26-50%<br>◯ 51-75%<br>◯ 76-100%

Final Placing of JV Water Polo Team at State Tournament	◯ 1<br>◯ 2<br>◯ 3<br>◯ 4<br>◯ 5<br>◯ 6<br>◯ 7<br>◯ 8<br>◯ 9<br>◯ 10th or lower<br>◯ No State Tournament
Final Placing of Varsity Water Polo Team at State Tournament	◯ 1<br>◯ 2<br>◯ 3<br>◯ 4<br>◯ 5<br>◯ 6<br>◯ 7<br>◯ 8<br>◯ 9<br>◯ 10th or lower<br>◯ No State Tournament
Knowing what you do now, was it the right decision to play this season?	◯ Yes<br>◯ No
Do you have any general thoughts about what you learned, what advice you would pass along?	__________________________________

College Water Polo Survey

Please complete the survey below.

Thank you!

Do you coach the men's team, women's team, or both?	◯ Men's Team<br>◯ Women's Team<br>◯ Both Men's and Women's Teams
Number of Athletes on Men's Team Who Participated in Activities this Spring	________________________
Number of Men's Practices This Spring	________________________
Number of Men's Games This Spring	________________________
Number of Men's Team COVID-19 Cases	________________________
Do you believe these were contracted because of in-pool water polo activities? (For instance, two players live together, and they both contracted COVID-19. You might think that this was related to them living together, not playing polo. Alternatively, two teammates contract COVID, that have their primary contact during the sport.)	◯ Yes<br>◯ No<br>◯ Uncertain
Please elaborate on any additional details of cases which would be helpful to know (e.g. The center contracted COVID-19, and then two days later, the center defender did)	______________________________
Did any male athletes on your team need to quarantine for contact with players on opposing team who developed COVID-19?	◯ Yes<br>◯ No
How many male athletes were quarantined due to exposure with opponent? (If an athlete needed to quarantine twice for exposures in two different games, please count that as 2. So, if you have team of 10 players, who all had to quarantine twice, please put 20)	________________________
How many of male athletes quarantined due to exposure with COVID-19 opponent ended up testing positive for COVID-19 during their quarantine?	________________________
Of all male athletes who contracted COVID-19, how many were symptomatic (had any symptoms, including fever, shortness of breath, cough, loss of taste or smell, vomiting, etc.)?	________________________

Of all male athletes who contracted COVID-19, how many athletes required longer than one week after quarantine ended to return to their baseline level of fitness?	__________________
Frequency of Men's team COVID-19 Routine Testing	◯ Daily<br>◯ Four Times Per Week<br>◯ Three Times Per Week<br>◯ Two Times Per Week<br>◯ Weekly<br>◯ Less than Once Per Week
By end of men's season, estimated percent of men's team fully-vaccinated? (Two weeks after receiving second Moderna or Pfizer vaccine, or two weeks after receiving J&J vaccine)	◯ < 25%<br>◯ 25-50%<br>◯ 51-75%<br>◯ 76-99%<br>◯ 100%
Number of Athletes on Women's Team Who Participated in Activities This Spring	__________________
Number of Women's Practices This Spring	__________________
Number of Women's Games This Spring	__________________
Number of Women's Team COVID-19 Cases	__________________
Do you believe these were contracted because of in-pool water polo activities? (For instance, two players live together, and they both contracted COVID-19. You might think that this was related to them living together, not playing polo. Alternatively, two teammates contract COVID, that have their primary contact during the sport.)	◯ Yes<br>◯ No<br>◯ Uncertain
Please elaborate on any additional details of cases which would be helpful to know (e.g. The center contracted COVID-19, and then two days later, the center defender did)	______________________
Did any female athletes on your team need to quarantine for contact with players on opposing team who developed COVID-19?	◯ Yes<br>◯ No
How many female athletes were quarantined? (If an athlete needed to quarantine twice for exposures in two different games, please count that as 2. So, if you have team of 10 players, who all had to quarantine twice, please put 20)	__________________
How many of female athletes quarantined due to exposure with COVID-19 opponent ended up testing positive for COVID-19 during their quarantine?	__________________

Of all female athletes who contracted COVID-19, how many were symptomatic (had any symptoms, including fever, shortness of breath, cough, loss of taste or smell, vomiting, etc.)?	________________________
Of all female athletes who contracted COVID-19, how many athletes required longer than one week after quarantine ended to return to their baseline level of fitness?	________________________
Frequency of Women's team COVID-19 Routine Testing	◯ Daily<br>◯ Four Times Per Week<br>◯ Three Times Per Week<br>◯ Two Times Per Week<br>◯ Weekly<br>◯ Less than Once Per Week
By end of women's season, estimated percent of women's team fully-vaccinated? (Two weeks after receiving second Moderna or Pfizer vaccine, or two weeks after receiving J&J vaccine)	◯ < 25%<br>◯ 25-50%<br>◯ 51-75%<br>◯ 76-99%<br>◯ 100%
